# Supplementary material for: Visual Deprivation Alters Functional Connectivity of Neural Networks for Voice Recognition: A Resting-State fMRI Study
Source: Brain Sci. 2023 Apr 7;13(4):636. doi: 10.3390/brainsci13040636 (PMC10136943; doi:10.3390/brainsci13040636)
Supplement: Supplementary file 1 [file brainsci-13-00636-s001.zip › brainsci-2246483-supplementary.pdf]

Supplementary Materials

Table S1. Characteristics of the early blind participants.

Subject	Age	Gender	Handedness	Residual vision	Onset of blindness	Cause of blindness
EB1	20	M	R	None	4 yrs	Retinal hemorrhage
EB2	22	M	R	None	Birth	Oxygen intoxication
EB3	35	M	R	None	Birth	Unknown congenital eye condition
EB4	22	F	R	Minimal LP	Birth	Retinal detachment
EB5	20	M	R	Minimal LP	Birth	Optic nerve atrophy
EB6	20	M	R	None	Birth	Glaucoma
EB7	25	F	R	Minimal LP	Birth	Retinal detachment
EB8	20	M	R	None	4 yrs	Unknown congenital eye condition
EB9	21	M	R	Minimal LP	Birth	Amblyopia
EB10	23	M	R	None	0~3 yrs	Glaucoma
EB11	26	M	R	Minimal LP	Birth	Optic nerve atrophy
EB12	18	M	R	Minimal LP	Birth	Optic nerve atrophy
EB13	18	M	R	Minimal LP	Birth	Cataract
EB14	23	F	R	Minimal LP	Birth	Optic nerve atrophy
EB15	21	M	R	None	Birth	Aplasia of macula
EB16	24	F	R	None	Birth	Retinoblastoma
EB17	26	M	R	None	0~2 yrs	Optic nerve atrophy
EB18	24	M	R	None	Birth	Microphthalmia
EB19	22	M	R	Minimal LP	Birth	Cataract
EB20	21	F	R	Minimal LP	Birth	Angiomatosis

Note: Gender (M, male; F, female), handedness (R, right-handed). The onset of blindness is defined as the absence of vision with at most minimal light perception.

Abbreviations: EB, early blind; LP, light perception; yrs, years.

Table S2. Region coordinates for defined regions of interest in the left hemisphere.

ROI	Region	*x*	*y*	*z*	Literature
L.TVAp	posterior superior temporal gyrus	-46	-38	2	Pernet et al., *NeuroImage*, 2015
L.TVAa	anterior superior temporal sulcus	-55	-8	-3	Pernet et al., *NeuroImage*, 2015
L.AMY	amygdala	-21	-5	-16	Sergerie et al., *Neuroscience and Biobehavioral Reviews*, 2008
L.IFG	inferior frontal gyrus (triangularis)	-48	14	29	Blank et al., *Neuroscience and Biobehavioral Reviews*, 2014
L.FFA	fusiform gyrus	-38	-49	-22	Blank et al., *Neuroscience and Biobehavioral Reviews*, 2014
L.OFA	middle occipital gyrus	-30	-90	12	Blank et al., *Neuroscience and Biobehavioral Reviews*, 2014

Abbreviations: L, left hemisphere; TVAp, posterior "temporal voice areas"; TVAa, anterior "temporal voice areas"; AMY, amygdala; IFG, inferior frontal gyrus; FFA, fusiform face area; OFA, occipital face area.

Table S3. The results of group comparisons between the sighted and blind participants in the strength of functional connectivity.

**Functional Connectivity**	***t*-value**	***p***	***p*-FDR**	***d***
TVAp-TVAa	0.487	0.629	0.726	0.151
TVAp-AMY	-2.947	0.005	0.027	0.911
TVAp-IFG	-0.752	0.457	0.623	0.232
TVAp-FFA	0.243	0.809	0.809	0.075
TVAp-OFA	0.400	0.692	0.741	0.124
TVAa-AMY	-2.565	0.014	0.030	0.793
TVAa-IFG	-1.034	0.307	0.461	0.320
TVAa-FFA	2.599	0.013	0.030	0.803
TVAa-OFA	2.720	0.010	0.029	0.840
AMY-IFG	-2.794	0.008	0.029	0.863
AMY-FFA	-0.551	0.585	0.726	0.170
AMY-OFA	-1.470	0.149	0.249	0.454
IFG-FFA	-3.214	0.003	0.019	0.993
IFG-OFA	-4.702	< .001	< .001	1.453
FFA-OFA	1.759	0.086	0.162	0.544

Abbreviations: TVAp, posterior “temporal voice areas”; TVAa, anterior “temporal voice areas”; AMY, amygdala; IFG, inferior frontal gyrus; FFA, fusiform face area; OFA, occipital face area.

Table S4. The results of group comparisons between the sighted control and early blind subjects in the strength of functional connectivity in the left hemisphere.

Functional Connectivity	*t*-value	*p*	*p*-FDR
L.TVAp-L.TVAa	0.640	0.526	0.617
L.TVAp-L.AMY	0.225	0.823	0.823
L.TVAp-L.IFG	-0.693	0.492	0.617
L.TVAp-L.FFA	0.839	0.407	0.610
L.TVAp-L.OFA	1.833	0.074	0.171
L.TVAa-L.AMY	-0.565	0.575	0.617
L.TVAa-L.IFG	-0.564	0.576	0.617
L.TVAa-L.FFA	1.181	0.244	0.407
L.TVAa-L.OFA	1.420	0.163	0.306
L.AMY-L.IFG	-2.408	0.021	0.078
L.AMY-L.FFA	-3.061	0.004	0.020
L.AMY-L.OFA	-1.797	0.080	0.171
L.IFG-L.FFA	-3.941	< .001	0.005
L.IFG-L.OFA	-3.571	0.001	0.007
L.FFA-L.OFA	-2.301	0.027	0.080

Abbreviations: L, left hemisphere; TVAp, posterior "temporal voice areas"; TVAa, anterior "temporal voice areas"; AMY, amygdala; IFG, inferior frontal gyrus; FFA, fusiform face area; OFA, occipital face area.

Table S5. Correlation of voice recognition accuracy and the strength of functional connectivity in the sighted.

r	**CN-GP**	**CN-DP**	**JP-GP**	**JP-DP**
TVAp-TVAa	0.079	0.118	0.064	0.014
TVAp-AMY	.471*	.436*	0.268	0.144
TVAp-IFG	0.015	-0.204	-0.020	-0.046
TVAp-FFA	.640**	0.210	0.310	-0.043
TVAp-OFA	0.419	0.234	0.332	0.078
TVAa-AMY	.441*	0.213	0.136	0.080
TVAa-IFG	0.058	-0.080	-0.184	-0.317
TVAa-FFA	.484*	0.189	0.171	0.275
TVAa-OFA	.502*	0.246	0.180	0.140
AMY-IFG	0.048	0.216	-0.141	-0.182
AMY-FFA	-0.315	-0.123	0.265	0.395
AMY-OFA	0.185	0.133	0.171	0.194
IFG-FFA	-0.230	-0.073	0.107	0.044
IFG-OFA	-0.090	-0.037	-0.004	0.064
FFA-OFA	0.374	0.253	0.046	-0.052

Note: Significance indicated by * (p uncorrected < .05), ** (p uncorrected < .01).

Abbreviations: TVAp, posterior "temporal voice areas"; TVAa, anterior "temporal voice areas"; AMY, amygdala; IFG, inferior frontal gyrus; FFA, fusiform face area; OFA, occipital face area; CN, Chinese condition; JP, Japanese condition; GP, generalization phase; DP, delayed memory phase.

Table S6. Correlation of voice recognition accuracy and the strength of functional connectivity in the blind.

r	**CN-GP**	**CN-DP**	**JP-GP**	**JP-DP**
TVAp-TVAa	-0.196	-0.063	-0.325	-0.256
TVAp-AMY	-0.286	-0.319	-0.320	-0.039
TVAp-IFG	-0.163	-0.120	-0.151	-0.162
TVAp-FFA	-0.014	0.070	0.048	-0.115
TVAp-OFA	-0.049	0.046	-0.083	-0.023
TVAa-AMY	-0.265	-0.169	-0.308	-0.075
TVAa-IFG	-0.009	0.125	0.068	0.079
TVAa-FFA	0.162	0.174	0.006	0.029
TVAa-OFA	0.044	0.171	0.058	-0.007
AMY-IFG	0.052	-0.078	-0.138	-0.150
AMY-FFA	.607**	.560*	.544*	.765**
AMY-OFA	.468*	.532*	0.372	.557*
IFG-FFA	0.031	-0.010	-0.068	0.139
IFG-OFA	-0.285	-0.126	-0.150	-0.123
FFA-OFA	.509*	.461*	0.231	0.294

Note: Significance indicated by * (p uncorrected < .05), ** (p uncorrected < .01).

Abbreviations: TVAp, posterior "temporal voice areas"; TVAa, anterior "temporal voice areas"; AMY, amygdala; IFG, inferior frontal gyrus; FFA, fusiform face area; OFA, occipital face area; CN, Chinese condition; JP, Japanese condition; GP, generalization phase; DP, delayed memory phase.

Table S7. The comparisons of correlations between the sighted control and early blind groups

p	CN-GP	CN-DP	JP-GP	JP-DP
TVAp-TVAa	0.406	0.586	0.229	0.409
TVAp-AMY	.016*	.017*	0.069	0.582
TVAp-IFG	0.591	0.796	0.692	0.725
TVAp-FFA	.021*	0.668	0.414	0.828
TVAp-OFA	0.138	0.564	0.200	0.762
TVAa-AMY	.026*	0.246	0.173	0.642
TVAa-IFG	0.841	0.538	0.446	0.222
TVAa-FFA	0.275	0.963	0.618	0.448
TVAa-OFA	0.128	0.814	0.711	0.658
AMY-IFG	0.990	0.373	0.993	0.922
AMY-FFA	.002**	.024*	0.311	0.077
AMY-OFA	0.337	0.169	0.514	0.196
IFG-FFA	0.427	0.850	0.599	0.774
IFG-OFA	0.543	0.788	0.659	0.574
FFA-OFA	0.614	0.472	0.571	0.288

Note: Significance indicated by * (p uncorrected < .05), ** (p uncorrected < .01).

Abbreviations: TVAp, posterior "temporal voice areas"; TVAa, anterior "temporal voice areas"; AMY, amygdala; IFG, inferior frontal gyrus; FFA, fusiform face area; OFA, occipital face area; CN, Chinese condition; JP, Japanese condition; GP, generalization phase; DP, delayed memory phase.
